# Subjective health and psychosomatic complaints of children and adolescents in Germany: Results of the HBSC study 2009/10 – 2022

**DOI:** 10.25646/11868

**Published:** 2024-03-04

**Authors:** Franziska Reiß, Steven Behn, Michael Erhart, Lisa Strelow, Anne Kaman, Veronika Ottová-Jordan, Ludwig Bilz, Irene Moor, Ulrike Ravens-Sieberer, for the HBSC Study Group Germany

**Affiliations:** 1 University Medical Center Hamburg-Eppendorf, Center for Psychosocial Medicine, Department of Child and Adolescent Psychiatry, Psychotherapy and Psychosomatics, Research Section Child Public Health, Hamburg, Germany; 2 Alice Salomon University of Applied Sciences Berlin, Health and Rehabilitation Science, Berlin, Germany; 3 Brandenburg University of Technology Cottbus-Senftenberg, Institute of Health, Cottbus, Germany; 4 Martin Luther University Halle-Wittenberg, Medical Faculty, Interdisciplinary Centre for Health Sciences (PZG), Institute of Medical Sociology, Halle (Saale), Germany

**Keywords:** CHILDREN, ADOLESCENTS, MENTAL HEALTH, SUBJECTIVE HEALTH, LIFE SATISFACTION, PSYCHOSOMATIC COMPLAINTS, PREVALENCES, SCHOOLS, HBSC, SURVEY, GERMANY

## Abstract

**Background:**

Subjective health and well-being are important health indicators in childhood and adolescence. This article shows current results and trends over time between 2009/10 and 2022.

**Methods:**

The Health Behaviour in School-aged Children (HBSC) study examined subjective health, life satisfaction and psychosomatic complaints of N = 21,788 students aged 11 to 15 years in the school years 2009/10, 2013/14, 2017/18 and in the calendar year 2022. Multivariate regression analyses show the associations between sociodemographic characteristics and well-being in 2022, as well as trends since 2009/10.

**Results:**

The majority of children and adolescents indicate a good subjective health and high life satisfaction. About half of the girls and one third of the boys report multiple psychosomatic health complaints, with a clear increase over time. Older adolescents, girls and gender diverse adolescents are at an increased risk of poor well-being. Subjective health and life satisfaction varied between 2009/10 and 2022, with a significant deterioration between 2017/18 and 2022.

**Conclusions:**

The high proportion of children and adolescents with psychosomatic complaints, as well as the observed gender and age differences, underline the need for target group-specific prevention, health promotion and continuous health monitoring.

## 1. Introduction

It is not only physical health that is important for children and adolescents to grow up healthy, but also their mental well-being. A look at the last decade and the present shows that several societal crises, such as the COVID-19 pandemic, the financial and energy crisis and the war in Ukraine, have (temporarily) changed societal coexistence. This has had an impact on the everyday lives of children and adolescents, affecting their health and well-being [1].

In line with the World Health Organization’s (WHO) definition of health, great importance is attributed to social and psychological well-being in addition to complete physical health. Subjective well-being has become an indispensable construct in public health research. Thereby, self-rated health, life satisfaction and psychosomatic complaints are commonly used as indicators of well-being [2, 3]. Good well-being is important throughout the entire life course, whereby impairments in health and well-being at an early age have negative effects into adulthood [4]. Good mental health and well-being are also closely related to health behaviour and healthcare utilisation, which in turn is related to the burden of disease (morbidity) and mortality [5–7].

**HBSC 2022**

**Data holder:** HBSC Study Group Germany

**Objective:** The aim of the study is to analyse the health and health behaviour of students. Continuous health monitoring through the HBSC study contributes to informing decision-makers in policy and practice about the current fields in prevention and health promotion in childhood and adolescence. A particular focus is on the influencing factors and the social contexts of health in the young generation.

**Study design:** Cross-sectional survey by written questionnaire every four years

**Population:** Students with average ages 11, 13, and 15

**Sampling:** Observation units are schools and the class groups clustered within them. From the population of all state general education schools in Germany, a cluster sample was drawn. In order to obtain a representative estimate (close to the distribution of the population), school size and the percentage distribution of students were included in the sampling, stratified by school type and federal state (Probability Proportional to Size (PPS) design).

**Data collection period:** March – November 2022

**Sample size:**

**2022:** 6,475 students

**All four survey cycles (2009/10 – 2022):** 21,788 students

**HBSC survey cycles:**

**Included in the articles in this issue of the Journal of Health Monitoring:**

▶ 2009/10
▶ 2013/14
▶ 2017/18
▶ 2022

More information can be found at https://hbsc-germany.de/ (German)

An earlier trend study by Ottova et al. [8] showed that students in Germany rated their health positively overall between 2002 and 2010. During this period, an improvement in self-rated health and a decrease in multiple recurrent health complaints were observed [8]. These findings have been confirmed in other international studies [9], for example the increase in life satisfaction between 2002 and 2010, particularly in Western European countries such as Austria, Denmark, Switzerland and Finland [10].

The outbreak of the COVID-19 pandemic in 2020 had a negative impact on the positive developments in health-related quality of life, life satisfaction, and mental health in Germany that had been observed over the previous two decades [11, 12]. As children and adolescents are in a vulnerable developmental period, they were particularly affected by the COVID-19 pandemic and the associated containment measures. Thereby, the loss of social contacts and increased family conflicts were particularly stressful for them [13]. As a result of the COVID-19 pandemic, international literature reviews and meta-analyses have reported increases in worries, anxiety, sleep problems and depressive symptoms in children and adolescents [14–18].

Research findings often indicate gender and age differences in self-rated health and well-being, as well as differences by migration background. Even before the COVID-19 pandemic, girls reported lower subjective well-being, lower life satisfaction and more psychosomatic complaints than boys [8]. In the context of the COVID-19 pandemic, girls also showed more symptoms of anxiety, depression and stress than boys [16]. In terms of age differences, adolescents were more likely to report mental health problems than children [11, 16]. Previous studies have reported mixed findings on the association between migration background and health [19].

Newest studies suggest that current societal crises contribute to further stress [20, 21]. Against this background, the question arises how subjective health, life satisfaction and psychosomatic health complaints have changed in adolescents in the period from before and towards the end of the coronavirus pandemic. The analysis and presentation of prevalences and trends is based on representative data and provides valuable information for the identification of needs, target group-specific interventions and the development of political and practical measures.

The aim of this study is to present the current prevalences of self-rated health, life satisfaction and psychosomatic health complaints in 11-, 13- and 15-year-old children and adolescents in Germany. In addition, health trends for the period from 2009/10 to 2022 are presented, taking into account age and gender differences. The research questions are as follows:

▶ What are the current prevalences of subjective health, life satisfaction and psychosomatic health complaints among students?
▶ What are the trends in subjective health, life satisfaction and psychosomatic health complaints for 11-, 13- and 15-year-olds between 2009/10 and 2022?
▶ What are the differences in subjective health, life satisfaction and psychosomatic complaints by age and gender during the period mentioned?
▶ What implications do these findings have for prevention and health promotion?

## 2. Methods

### 2.1 Sample design and study implementation

The Health Behaviour in School-aged Children (HBSC) study is designed as a cross-sectional study that takes place every four years in a school setting and surveys students aged around 11, 13 and 15 (mean deviation of 0.5 years). In Germany, these age groups mainly comprise grades 5, 7, and 9. Students at general education schools in all 16 federal states in Germany have been surveyed in the school years 2009/10, 2013/14, 2017/18 and in the calendar year 2022 as part of the HBSC study. The schools contacted for participation were drawn as a cluster sample from the population of all state general education schools in Germany. In order to obtain a representative estimate (close to the distribution of the population), school size and the percentage distribution of students were included in the sampling, stratified by school type (Probability Proportional to Size (PPS) design).

The HBSC study is conducted by means of a questionnaire, which students complete themselves. The study has been approved by the responsible ministries or state education authorities in all federal states (except North Rhine-Westphalia, as the decision of participation lies within the schools in this federal state).

Four survey cycles of the HBSC study Germany were analysed for the present study. In addition to the current survey in 2022 (n = 6,475), three further surveys were included in the following school years: 2009/10 (n = 5,005), 2013/14 (n = 5,961) and 2017/18 (n = 4,347). All data sets were standardised and adjusted by the international HBSC consortium so that the age groups are comparable. Further information on the HBSC study and the methodology can be found in the article by Winter & Moor et al. [23] in this issue of the Journal of Health Monitoring.

### 2.2 Instruments

Subjective health and well-being were assessed by using the indicators self-rated health, life satisfaction and psychosomatic health complaints.

Self-rated health comprises an individual’s perception and judgement of their own health [24]. Students were asked how they rate their health, with the response options ‘excellent’, ‘good’, ‘fair’ and ‘poor’. The upper categories ‘excellent’ and ‘good’ and the lower categories ‘fair’ and ‘poor’ were summarised as ‘rather good’ and ‘rather poor’ health, respectively.

Life satisfaction captures the evaluation of one’s life as an expression of subjective well-being and was assessed using the Cantril Ladder [25]. The participants were asked to use an 11-point visual analogue scale in the form of a ladder to indicate their current life satisfaction. The top of the ladder represented the ‘best possible life’ (10 points) and the bottom represented the ‘worst possible life’ (0 points). In accordance with the HBSC standard, responses were dichotomised for analysis into ‘low life satisfaction’ (0 to 5 points) and ‘high life satisfaction’ (6 to 10 points).

Psychosomatic health complaints were assessed with the HBSC Symptom Checklist (HBSC-SCL) [26]. Using a five-point answer scale ranging from ‘about every day’ to ‘rarely or never’, students were asked to indicate how often they had experienced headache, stomachache, backache, feeling low, irritability/bad mood, nervousness, difficulties falling asleep and drowsiness/dizziness during the past six months. Responses ranged from ‘almost every day’, ‘several times a week’, ‘almost every week’, ‘about once a month’ to ‘rarely or never’. If two or more of these complaints occurred at least once a week, they were referred to as ‘multiple recurrent complaints’.

Sociodemographic characteristics include age, gender and migration background. Gender was recorded in the 2022 survey year using the three options ‘girl’, ‘boy’ or ‘diverse’. In the previous survey cycles, gender was recorded in binary form (girl, boy). For the trend analyses, participants who did not specify their gender or classified themselves as diverse were excluded from the gender-specific analyses. The age was determined at the time of the survey using the information provided by the students on their month and year of birth and summarised with a deviation of +/- 0.5 years into the age categories ‘11 years’, ‘13 years’ and ‘15 years’. The migration background was assessed by asking about the students’ own country of birth and the country of birth of their parents. Adolescents were classified as having a one-sided migration background if one of their parents was not born in Germany. A two-sided migration background was classified if the adolescents were not born in Germany and at least one parent was not born in Germany, or if both parents immigrated or were not born in Germany. Further information can be found in Moor et al. [27].

### 2.3 Statistical methods

Statistical analyses were performed using the data from the German HBSC survey cycles 2009/10 until the calendar year 2022.

Descriptive results for subjective health, life satisfaction and psychosomatic health complaints are presented as prevalences or proportions stratified by gender (girls, boys, diverse) and age (11-, 13- and 15-year-olds). Differences in the number of respondents between health indicators are due to differences in the number of missing values. The correlation between sociodemographic characteristics (gender, age and migration background) and the indicators of subjective health, life satisfaction and psychosomatic health complaints was calculated using multivariate logistic regression models, adjusting for all other included variables. Results are presented as odds ratios (OR) and 95 % confidence intervals (CI).

The time trends of the health indicators were described for the four survey cycles 2009/10 to 2022 using proportions (95 % CI). In addition, binary logistic regressions with OR and 95 % CI were calculated, using the four survey cycles (with 2009/10 as the reference for the first measurement point), gender (girls and boys), age (11-, 13- and 15-year-olds) and migration background as independent variables. The prevalences and effect estimates of the regression models are reported weighted.

A weighting factor was created for all survey cycles to ensure nationwide sample representativeness. This equalises different participation rates in the federal states and school types so that the distribution corresponds to the population. Due to the weighting, all three age categories and the binary gender categories of girls and boys are included in the analyses in equal parts from the 2017/18 survey cycle onwards. In the 2022 HBSC survey cycle, gender was not recorded exclusively in binary form for the first time, with 1.7 % of respondents indicating the category gender diverse. This was taken into account in the weighting of the 2022 data, while girls and boys were weighted equally (49.2 % each; participants who did not specify their gender were excluded). Further details on the weighting of the data can be found in the article by Winter & Moor et al. [23]. All analyses were performed taking into account the weighting variables; absolute figures refer to unweighted data. All analyses were carried out using IBM SPSS Statistics 28.

## 3. Results

A total of N = 21,788 students aged 11, 13 and 15 years participated in the HBSC study in the survey period 2009/10 to 2022 (2009/10: N = 5,005, 2013/14: N = 5,961, 2017/18: N = 4,347 and 2022: N = 6,475). Of these, 51 % were girls (n = 11,066). The current results for 2022 and trends from 2009/10 to 2022 are shown below.

### 3.1 Current results for the 2022 survey cycle

The 2022 HBSC survey included N = 6,475 participants, of whom n = 3,074 were boys (47.5 %), n = 3,258 girls (50.3 %), n = 112 gender diverse (1.7 %), and n = 31 (0.5 %) had missing gender information. 38.0 % of the respondents had a migration background. The distribution across the age groups of 11-, 13- and 15-year-olds was balanced (33.7 %, 34.0 %, and 32.1 %, respectively).

**Figure 1:**
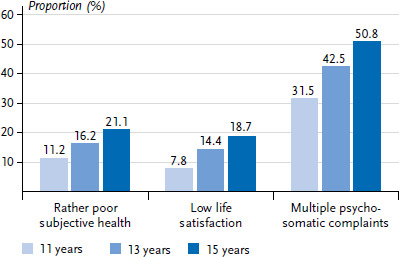
**Proportion of students with rather poor subjective health, low life satisfaction and multiple psychosomatic complaints by age (N = 6,465, n = 2,164 11-year-olds, n = 2,177 13-year-olds, n = 2,124 15-year-olds)** Source: HBSC Germany 2022

**Figure 2:**
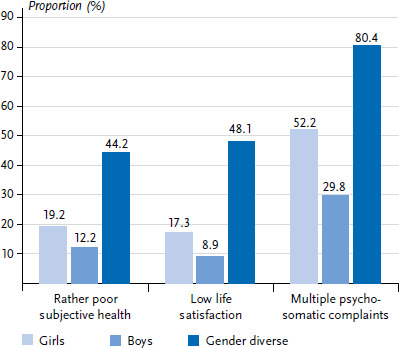
**Proportion of students with rather poor subjective health, low life satisfaction and multiple psychosomatic complaints by gender (N = 6,444, n = 3,258 girls, n = 3,074 boys, n = 112 gender diverse)** Source: HBSC Germany 2022

Figure 1 and Figure 2 show the results for self-rated health, life satisfaction and psychosomatic complaints by age and gender in 2022. Overall, the majority of students reported ‘rather good’ health, while 16.1 % reported ‘rather poor’ health. Older adolescents, girls and diverse adolescents were less likely to report ‘rather good’ health than younger adolescents and boys, with diverse adolescents reporting the worst health perception. In contrast, there was little difference in self-rated health by migration background.

The majority of adolescents reported a high level of life satisfaction (86.3 %), with boys showing the highest overall life satisfaction compared to girls and gender diverse respondents (91.1 % vs. 82.7 % vs. 51.9 %). Boys’ life satisfaction remained largely stable across the age groups, while girls’ life satisfaction decreased with increasing age. The life satisfaction of young people who describe themselves as gender diverse increased between the ages of 11 and 13, but fell again at age 15. Students with a two-sided migration background were more likely to report lower life satisfaction than those without a migration background (20.0 % vs. 11.1 %).

In 2022, a total of 41.7 % of all respondents reported multiple psychosomatic health complaints. These were reported significantly more often by girls and gender diverse respondents than by boys (52.2 % and 80.4 %, respectively, compared with 29.8 %). This gender difference increased with age, with about half of 15-year-olds suffering from multiple psychosomatic complaints on a weekly basis. Children and adolescents with a one-sided or two-sided migration background were slightly more likely to report psychosomatic complaints than those without a migration background (44.1 % and 43.5 % vs. 40.5 %).

Table 1 shows the results of the multivariate logistic regression analysis. The odds of a ‘rather poor’ health perception were up to 2 times higher for 13- and 15-year-olds than for 11-year-olds. Compared with boys, girls had a 1.7-fold higher risk of a ‘rather poor’ health perception, while gender diverse participants had a 4.4-fold higher risk. There were no significant differences with regard to migration background. Overall, the model was able to explain 5.3 % of the variance in subjective health.

The odds of low life satisfaction were 1.9 and 2.5 times higher for 13- and 15-year-olds, respectively, than for 11-year-olds. Again, the odds of low life satisfaction were 2.2 times and 8.5 times higher for girls and gender diverse participants than for boys. Both one-sided and two-sided migration background were associated with a 1.4-fold and 1.9-fold increased risk of low life satisfaction, respectively. The variables explained 11.3 % of the variance in life satisfaction.

A slightly increased risk of multiple psychosomatic health complaints was found for students with a one-sided and a two-sided migration background compared to their peers without a migration background. 13- and 15-year-old students had a 1.6-fold and 2.2-fold higher risk of multiple psychosomatic complaints compared with 11-year-olds, respectively. Girls and gender diverse students had a 2.6-fold and 8.5-fold higher risk of psychosomatic health complaints, respectively, compared with boys. Overall, the model explained 11.5 % of the variance in multiple psychosomatic complaints.

### 3.2 Health trends from 2009/10 to 2022

**Figure 3:**
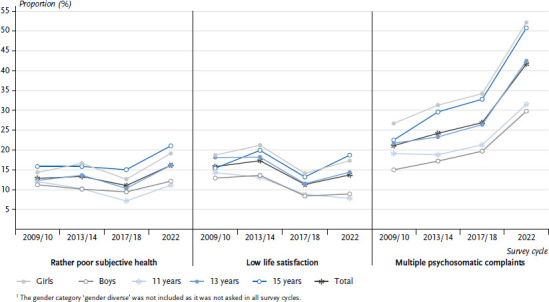
**Proportion of students with rather poor subjective health, low life satisfaction and multiple psychosomatic complaints by age, gender ^1^ and survey year** Source: HBSC Germany 2009/10, 2013/14, 2017/18, 2022

Figure 3 shows the descriptive trends over time for self-rated health, life satisfaction and multiple psychosomatic complaints. Compared to previous years, there was a significant deterioration in subjective health in the 2022, especially among 15-year-olds. There was also a more pronounced deterioration in self-rated health among girls (+ 6.5 %) compared to boys of the same age (+ 2.7 %) between 2017/18 and 2022.

Both in the overall analysis and between the gender and age groups, there was a deterioration in life satisfaction from 2009/10 to 2013/14, an improvement until 2017/18 and then a deterioration again to 2022. The exception was 11-year-olds, whose life satisfaction increased steadily over all survey cycles. Compared to boys, girls showed significantly lower levels of life satisfaction, with varying trends over the past ten years.

The occurrence of multiple psychosomatic health complaints increased overall in all survey cycles, with a significant rise between 2017/18 and 2022 (+ 14.8 %). In all four surveys, girls and older students were significantly more likely to have multiple psychosomatic complaints than boys and younger students. In addition, a higher increase in multiple psychosomatic complaints was observed with increasing age between 2017/18 and 2022 (11-year-olds: + 10.3 %, 13-year-olds: + 16.1 %, 15-year-olds: + 18.0 %).

Table 2 shows the results of the multivariate logistic regression for self-rated health, life satisfaction and psychosomatic health complaints over time from 2009/10 to 2022. It shows that the risk of having a rather poor self-rated health and multiple psychosomatic health complaints was significantly higher in 2022 than in the 2009/10 reference period. The trend analyses showed a significant improvement in life satisfaction in 2017/18 and 2022 compared to 2009/10. While subjective health was rated significantly better overall in 2017/18 compared to 2009/10, a significant deterioration was observed in 2022 compared to 2009/10.

There were significant health trends by gender over the whole time course, with girls having a 1.5 times higher risk of a rather poor self-rated health, a 1.8 times higher risk of low life satisfaction and a 2.3 times higher risk of multiple psychosomatic complaints compared to boys. It was also observed that 13- and 15-year-olds had a higher risk of low life satisfaction and multiple psychosomatic health complaints than 11-year-olds.

The trend analyses also showed that both a one-sided and a two-sided migration background were associated with an increased risk of rather poor subjective health, low life satisfaction and multiple psychosomatic health complaints.

Overall, 3.0 % of the variance in subjective health, 4.5 % of the variance in life satisfaction and 10.8 % of the variance in multiple psychosomatic complaints could be explained by the respective models.

## 4. Discussion

The fundamental goals of public health research include not only the monitoring of health, but also the improvement of population health and reduction of health inequalities [28]. The current results of the HBSC 2022 study show that the majority of children and adolescents have good subjective health and a high level of overall life satisfaction. This finding is consistent with the results of previous cycles of the HBSC study [29] and other population-based studies (KiGGS, BELLA) [30, 31], which also reported good to very good general health among children and adolescents. However, the current HBSC data also show that about half of the girls and one third of the boys reported multiple psychosomatic complaints such as headache, stomachache and difficulty falling asleep. For the first time, the 2022 HBSC survey also analysed gender diverse children and adolescents, who reported poorer subjective health, lower life satisfaction and more frequent psychosomatic complaints in all age groups. These findings are supported by international studies showing that non-binary youth are particularly affected by verbal and physical violence and general rejection [32–34]. With regard to age, older adolescents showed a deterioration in all indicators of subjective health and well-being. These findings are in line with previous national and international studies, which have also shown that girls and older adolescents are more likely to report poor health, multiple psychosomatic complaints and lower life satisfaction [16, 20, 29, 35]. One possible explanation for the gender differences could be an increased sensitivity to stress due to hormonal changes in girls during puberty [36]. Another explanation could be that girls are more strongly encouraged to express and communicate emotional feelings than boys as a result of socialisation [37].

Looking at the overall time trends from 2009/10 to 2022, both subjective health and life satisfaction show a fluctuating course, with the proportion of children and adolescents with rather poor subjective health and low life satisfaction increasing significantly in 2022 – compared to 2017/18. The trend in life satisfaction is consistent with the longitudinal results of Reiß et al. [11], who also analysed data from the COPSY and BELLA studies in addition to data from the HBSC study. The trend analyses also show that multiple psychosomatic complaints increased continuously between 2009/10 and 2022. Between 2017/18 and 2022, the proportion of students with such complaints increased the most clearly. This could be, among other factors, due to the effects of the COVID-19 pandemic. Even two years after the outbreak of the COVID-19 pandemic, these results illustrate the possible consequences of pandemic-related stress due to the drastic restrictions in children’s and adolescents’ daily lives and social relationships. The results are consistent with numerous international reviews and meta-analyses describing the negative effects of the pandemic on the well-being of adolescents not only at the beginning of the pandemic [38–43], but also as the pandemic progressed [1, 16]. An increase in symptoms of anxiety and depression among children and adolescents has also been reported at the national level during the pandemic [12, 44]. Thus, the changes and stress caused by the pandemic may explain the significant deterioration in psychosomatic complaints, which also highlights the need for action and for targeted prevention and intervention programmes in times of crisis. In addition, other studies show that almost a half of young people in Germany feel burdened by other crises, such as the climate crisis, the energy crisis or the war in Ukraine, and feel that their mental health is impaired [20, 21, 45]. One third of young people also report anxiety about the impact of these crises on their future [13], which in turn could have long-term consequences for their own health and is therefore relevant to public health. Although later studies, such as the nationwide COPSY study (‘COVID-19 and PSYchological Health’), show an improvement in health-related quality of life and life satisfaction over time, as well as a downward trend in mental health problems, they have not yet returned to pre-pandemic levels [13, 44].

###  

#### Strengths and limitations

The strength of the HBSC study lies in the high methodological quality achieved through the use of internationally validated measurement instruments, a large sample and a standardised procedure. Another strength of the study is that it captures the analysed indicators from the perspective of the children and adolescents themselves. The repeated cross-sectional surveys make it possible to depict age- and gender-related trends in the subjective health and well-being of children and adolescents for a comprehensive health monitoring. Due to the large number of European and non-European countries now participating in the HBSC study (www.hbsc.org), direct international comparisons will be possible in the future. One limitation is the cross-sectional design of the HBSC study, which means that it is not possible to draw conclusions about causal relationships and developmental trajectories. Due to the several months of data collection for the current HBSC study from March onwards, possible effects of the dynamic course of the COVID-19 pandemic are conceivable, e.g. that the Omicron wave of the winter and the associated restrictions at the beginning of spring had an even greater impact on subjective health and well-being than at a later survey date in the calendar year 2022.

#### Outlook

Girls and gender diverse students as well as older adolescents are a large, heterogeneous and vulnerable population that should be particularly addressed in mental health prevention and interventions. Existing policies should therefore be reviewed and adapted to address persistent age and gender differences. The marked declines in self-rated health and well-being of children and adolescents reported in this article reinforce the existing demands of the German Ethics Council [46] and the National Action Plan ‘New Opportunities for Children in Germany’ [47]. Both statements emphasise specific health promotion and the rapid expansion of resources for comprehensive psychological and therapeutic support and care for children and adolescents. In addition, continuous health monitoring of children and adolescents is recommended. Thereby, the present HBSC study can contribute as a valuable data source, which provides continuous up-to-date data and facilitates the investigation of further development processes and trend analyses.

## Key statement

- The majority of children and adolescents indicate a good overall subjective health and a high level of life satisfaction.
- Girls, gender diverse and older adolescents are more likely to report poor health, low life satisfaction or multiple psychosomatic complaints.
- The reported subjective health and life satisfaction deteriorated in 2022 compared to the survey periods 2009/10 to 2017/18.
- The occurrence of multiple psychosomatic complaints increased between 2009/10 and 2022, and reached a new high level in 2022.
- Target group-specific prevention and health promotion programmes are necessary to promote the subjective health and well-being of children and adolescents.

## Figures and Tables

**Table 1:** **Odds ratios and 95 % confidence intervals for rather poor subjective health, low life satisfaction and multiple psychosomatic complaints by age, gender and migration background (multivariate logistic regression model including all predictors)** Source: HBSC Germany 2022

	Rather poor subjective health (n = 6,338)		Low life satisfaction (n = 6,276)		Multiple psychosomatic complaints (n = 6,244)	
	OR	(95 % CI)	OR	(95 % CI)	OR	(95 % CI)
**Age**						
11 years (ref.)						
13 years	1.49	(1.24 – 1.79)^**^	1.87	(1.52 – 2.30)^**^	1.60	(1.40 – 1.83)^**^
15 years	2.03	(1.70 – 2.42)^**^	2.47	(2.02 – 3.02)^**^	2.23	(1.95 – 2.55)^**^
**Gender**						
Boys (ref.)						
Girls	1.66	(1.44 – 1.91)^**^	2.22	(1.86 – 2.60)^**^	2.64	(2.37 – 2.94)^**^
Gender diverse	4.44	(2.94 – 6.72)^**^	8.49	(5.59 – 12.88)^**^	8.50	(5.14 – 14.08)^**^
**Migration background**						
None (ref.)						
One-sided	1.00	(0.81 – 1.24)	1.39	(1.10 – 1.76)^**^	1.24	(1.05 – 1.46)^*^
Two-sided	0.87	(0.73 – 1.03)	1.87	(1.58 – 2.22)^**^	1.19	(1.05 – 1.36)^*^
Nagelkerke’s R^2^	0.053		0.113		0.115	

OR = odds ratio, CI = confidence interval, ref. = reference category,

^*^ p < 0.05,

^**^ p < 0.001

**Table 2:** **Odds ratios and 95 % confidence intervals for rather poor subjective health, low life satisfaction and multiple psychosomatic complaints by survey cycle, age, gender^1^ and migration background over the HSBC survey cycles 2009/10 to 2022** Source: HBSC Germany 2009/10, 2013/14, 2017/18, 2022

	Rather poor subjective health (n = 20,465)		Low life satisfaction (n = 20,332)		Multiple psychosomatic complaints (n = 20,274)	
	OR	(95 % CI)	OR	(95 % CI)	OR	(95 % CI)
**Survey cycle**						
2009/10 (ref.)						
2013/14	1.02	(0.91 – 1.15)	1.09	(0.98 – 1.21)	1.19	(1.40 – 1.83)^**^
2017/18	0.81	(0.71 – 0.92)^*^	0.63	(0.56 – 0.72)^**^	1.37	(1.24 – 1.51)^**^
2022	1.26	(1.13 – 1.40)^**^	0.75	(0.67 – 0.83)^**^	2.69	(2.46 – 2.94)^**^
**Age**						
11 years (ref.)						
13 years	1.44	(1.29 – 1.60)^**^	1.54	(1.39 – 1.71)^**^	1.39	(1.29 – 1.51)^*^
15 years	1.89	(1.71 – 2.10)^**^	1.66	(1.50 – 1.84)^**^	1.82	(1.68 – 1.97)^*^
**Gender**						
Boys (ref.)						
Girls	1.53	(1.41 – 1.66)^**^	1.80	(1.66 – 1.95)^**^	2.27	(2.13 – 2.43)^**^
**Migration background**						
None (ref.)						
One-sided	1.34	(1.18 – 1.52)^**^	1.41	(1.24 – 1.59)^**^	1.33	(1.20 – 1.47)^**^
Two-sided	1.16	(1.05 – 1.29)^*^	1.70	(1.54 – 1.87)^**^	1.33	(1.23 – 1.44)^**^
Nagelkerke’s R^2^	0.030		0.045		0.108	

OR = odds ratio, CI = confidence interval, ref. = reference category,

^*^ p < 0.05,

^**^ p < 0.001

^1^ The gender category ‘gender diverse’ was not included as it was not asked in all survey cycles.

## References

[1] Orban E Li L Gilbert M(2023) Mental health and quality of life in children and adolescents during the COVID-19 pandemic – A systematic review of longitudinal studies. Front Public Health 11:1275917 38259801 10.3389/fpubh.2023.1275917 PMC10800626

[2] Diener E Suh EM Lucas RE(1999) Subjective well-being: Three decades of progress. Psychol Bull 125(2):276–302

[3] Kiefer RA (2008) An integrative review of the concept of well-being. Holist nurs pract 22(5):244–252 18758272 10.1097/01.HNP.0000334915.16186.b2

[4] Sawyer SM Afifi RA Bearinger LH(2012) Adolescence: a foundation for future health. Lancet 379(9826):1630–1640 22538178 10.1016/S0140-6736(12)60072-5

[5] Latham K Peek CW (2013) Self-rated health and morbidity onset among late midlife U.S. adults. J Gerontol B Psychol Sci Soc Sci 68(1):107–116 23197340 10.1093/geronb/gbs104 PMC3605944

[6] Lorem G Cook S Leon DA(2020) Self-reported health as a predictor of mortality: A cohort study of its relation to other health measurements and observation time. Sci Rep 10(1):4886 32184429 10.1038/s41598-020-61603-0 PMC7078209

[7] Ul-Haq Z Mackay DF Pell JP (2014) Association between self-reported general and mental health and adverse outcomes: a retrospective cohort study of 19,625 Scottish adults. PLoS One 9(4):e93857 24705574 10.1371/journal.pone.0093857 PMC3976324

[8] Ottova V Hillebrandt D Ravens-Sieberer U(2012) Trends in der subjektiven Gesundheit und des gesundheitlichen Wohlbefindens von Kindern und Jugendlichen in Deutschland: Ergebnisse der Health Behaviour in School-aged Children (HBSC) Studie 2002 bis 2010. Gesundheitswesen 74(Suppl 1):15–24 10.1055/s-0032-1312640 22836886

[9] Ottová-Jordan V Smith ORF Augustine L(2015) Trends in health complaints from 2002 to 2010 in 34 countries and their association with health behaviours and social context factors at individual and macro-level. Eur J Public Health 25 (Suppl 2):83–89 25805796 10.1093/eurpub/ckv033

[10] Cavallo F Dalmasso P Ottová-Jordan V(2015) Trends in life satisfaction in European and North-American adolescents from 2002 to 2010 in over 30 countries. Eur J Public Health 25 (Suppl 2):80–82 25805795 10.1093/eurpub/ckv014

[11] Reiß F Kaman A Napp AK(2023) Epidemiologie seelischen Wohlbefindens von Kindern und Jugendlichen in Deutschland. Ergebnisse aus 3 Studien vor und während der COVID-19-Pandemie. Bundesgesundheitsbl 66(7):727–735 10.1007/s00103-023-03720-5 PMC10227816 37249582

[12] Schlack R Neuperd L Junker S(2023) Changes in the mental health in the children’s and adolescent population in Germany during the COVID-19 pandemic – Results of a rapid review. J Health Monit 8(S1):1–72. https://doi.org/10.25646/10761 (As at 21.12.2023) 10.25646/10761 PMC9936565 36818693

[13] Ravens-Sieberer U Kaman A Devine J(2023) Die COVID-19-Pandemie – Wie hat sie die Kinderpsyche beeinflusst? Monatsschr Kinderheilkd 171(7):608–614 10.1007/s00112-023-01775-x PMC10243683 37362308

[14] Haig-Ferguson A Cooper K Cartwright E(2021) Practitioner review: health anxiety in children and young people in the context of the COVID-19 pandemic. Behav Cogn Psychother 49(2):129–143 32829718 10.1017/S1352465820000636 PMC7503041

[15] Fegert JM Vitiello B Plener PL(2020) Challenges and burden of the Coronavirus 2019 (COVID-19) pandemic for child and adolescent mental health: a narrative review to highlight clinical and research needs in the acute phase and the long return to normality. Child Adolesc Psychiatry Ment Health 14, 20. https://doi.org/10.1186/s13034-020-00329-3 (As at 21.12.2023) 32419840 10.1186/s13034-020-00329-3 PMC7216870

[16] Wolf K Schmitz J (2023) Scoping review: longitudinal effects of the COVID-19 pandemic on child and adolescent mental health. Eur Child Adolesc Psychiatry. https://doi.org/10.1007/s00787-023-02206-8 (As at 21.12.2023) 10.1007/s00787-023-02206-8 PMC10119016 37081139

[17] Deng J Zhou F Hou W(2023) Prevalence of mental health symptoms in children and adolescents during the COVID-19 pandemic: A meta-analysis. Ann N Y Acad Sci 1520(1):53–73 36537131 10.1111/nyas.14947 PMC9880764

[18] Samji H Wu J Ladak A(2022) Review: Mental health impacts of the COVID-19 pandemic on children and youth – a systematic review. Child Adolesc Ment Health 27(2):173–189 34455683 10.1111/camh.12501 PMC8653204

[19] Koschollek C Bartig S Rommel A(2019) The health of children and adolescents with a migration background in Germany – Results of the cross-sectional KiGGS Wave 2 study. J Health Monit 4(3):7–28. https://doi.org/10.25646/6074 (As at 21.12.2023) 10.25646/6074 PMC8822254 35146251

[20] Ravens-Sieberer U Kaman A Erhart M(2022) Impact of the COVID-19 pandemic on quality of life and mental health in children and adolescents in Germany. Eur Child Adolesc Psychiatry 31(6):879–889 33492480 10.1007/s00787-021-01726-5 PMC7829493

[21] Schnetzer S Hurrelmann K (2022) Trendstudie: Jugend in Deutschland. Jugend im Dauerkrisenmodus – Klima, Krieg, Corona. https://simon-schnetzer.com/jugend-in-deutschland-trend-studie-sommer-2022/ (As at 21.12.2023)

[22] Inchley JC Stevens G Samdal O(2020) Enhancing Understanding of Adolescent Health and Well-Being: The Health Behaviour in School-aged Children Study. J Adolesc Health 66(6s):S3–S5 10.1016/j.jadohealth.2020.03.014 32446607

[23] Winter K Moor I Markert J(2024) Concept and methodology of the Health Behaviour in School-aged Children (HBSC) study – Insights into the current 2022 survey and trends in Germany. J Health Monit 9(1):99–117. www.rki.de/jhealthmonit-en (As at 04.03.2024) 10.25646/11878 PMC10977469 38559683

[24] Breidablik HJ Meland E Lydersen S (2008) Self-rated health in adolescence: a multifactorial composite. Scand J Public Health 36(1):12–20 18426780 10.1177/1403494807085306

[25] Cantril H (1965) The pattern of human concerns. Rutgers University Press New Brunswick, New Jersey, New Brunswick, New Jersey.

[26] Haugland S Wold B Stevenson J(2001) Subjective health complaints in adolescence: A cross-national comparison of prevalence and dimensionality. Eur J Public Health 11(1):4–10 11276570 10.1093/eurpub/11.1.4

[27] Moor I Winter K Bilz L(2020) The 2017/18 Health Behaviour in School-aged Children (HBSC) study – Methodology of the World Health Organization’s child and adolescent health study. J Health Monit 2020(3):88–102. 10.25646/6904 (As at 21.12.2023) PMC8734187 35146275

[28] Zukunftsforum Public Health (2021) Eckpunkte einer Public-Health-Strategie für Deutschland. Zukunftsforum Public Health. https://zukunftsforum-public-health.de/public-health-strategie/(As at 21.12.2023)

[29] Kaman A Ottová-Jordan V Bilz L(2020) Subjective health and well-being of children and adolescents in Germany – Cross-sectional results of the 2017/18 HBSC study. J Health Monit 2020(3):7–20. 10.25646/6899 (As at 21.12.2023) PMC8734126 35146270

[30] Poethko-Müller C Kuntz B Lampert T(2018) The general health of children and adolescents in Germany. Results of the cross-sectional KiGGS Wave 2 study and trends. J Health Monit 3(1):8–14. 10.17886/RKI-GBE-2018-021 (As at 21.12.2023) PMC8848783 35586181

[31] Otto C Reiss F Voss C(2021) Mental health and well-being from childhood to adulthood: design, methods and results of the 11-year follow-up of the BELLA study. Eur Child Adolesc Psychiatry 30(10):1559–1577 32918625 10.1007/s00787-020-01630-4 PMC8505294

[32] Day JK Perez-Brumer A Russell ST (2018) Safe Schools? Transgender Youth’s School Experiences and Perceptions of School Climate. J Youth Adolesc 47(8):1731–1742 29858740 10.1007/s10964-018-0866-x PMC7153781

[33] Klemmer CL Rusow J Goldbach J(2021) Socially Assigned Gender Nonconformity and School Violence Experience Among Transgender and Cisgender Adolescents. J Interpers Violence 36(15/16):NP8567–NP8589 31023178 10.1177/0886260519844781

[34] Sares-Jäske L Czimbalmos M Majlander S(2023) Gendered Differences in Experiences of Bullying and Mental Health Among Transgender and Cisgender Youth. J Youth Adolesc 52(8):1531–1548 37199852 10.1007/s10964-023-01786-7 PMC10276116

[35] Chen X Cai Z He J(2020) Gender Differences in Life Satisfaction Among Children and Adolescents: A Meta-analysis. J Happiness Stud 21(6):2279–2307

[36] Henkens JHD Kalmijn M de Valk HAG (2022) Life Satisfaction Development in the Transition to Adulthood: Differences by Gender and Immigrant Background. J Youth Adolesc 51(2):305–319 35024977 10.1007/s10964-021-01560-7 PMC8828595

[37] Lyyra N Välimaa R Tynjälä J (2018) Loneliness and subjective health complaints among school-aged children. Scand J Public Health 46(20_suppl):87–93 29552967 10.1177/1403494817743901

[38] Ma L Mazidi M Li K(2021) Prevalence of mental health problems among children and adolescents during the COVID-19 pandemic: A systematic review and meta-analysis. J Affect Disord 293:78–89 34174475 10.1016/j.jad.2021.06.021 PMC9711885

[39] Meade J (2021) Mental Health Effects of the COVID-19 Pandemic on Children and Adolescents A Review of the Current Research. Pediatr Clin North Am 68(5):945–959 34538305 10.1016/j.pcl.2021.05.003 PMC8445752

[40] Meherali S Punjani N Louie-Poon S(2021) Mental Health of Children and Adolescents Amidst COVID-19 and Past Pandemics: A Rapid Systematic Review. Int J Environ Res Public Health 18(7):3432 33810225 10.3390/ijerph18073432 PMC8038056

[41] Panchal U de Pablo GS Franco M(2023) The impact of COVID-19 lockdown on child and adolescent mental health: systematic review. Eur Child Adolesc Psychiatry 32(7):1151–1177 34406494 10.1007/s00787-021-01856-w PMC8371430

[42] Panda PK Gupta J Chowdhury SR(2021) Psychological and Behavioral Impact of Lockdown and Quarantine Measures for COVID-19 Pandemic on Children, Adolescents and Caregivers: A Systematic Review and Meta-Analysis. J Trop Pediatr 67(1): fmaa122 33367907 10.1093/tropej/fmaa122 PMC7798512

[43] Kauhanen L Wan Mohd Yunus WMA Lempinen L(2023) A systematic review of the mental health changes of children and young people before and during the COVID-19 pandemic. Eur Child Adolesc Psychiatry 32(6):995–1013 35962147 10.1007/s00787-022-02060-0 PMC9373888

[44] Ravens-Sieberer U Devine J Napp AK(2023) Three years into the pandemic: results of the longitudinal German COPSY study on youth mental health and health-related quality of life. Front Public Health 11:1129073 37397777 10.3389/fpubh.2023.1129073 PMC10307958

[45] Peter F Dohm L Krimmer M (2023) Psychische Konsequenzen der Klimakrise. Monatsschr Kinderheilkd 171(2):130–137

[46] Deutscher Ethikrat (2022) Ad-hoc-Empfehlung: Pandemie und psychische Gesundheit. Aufmerksamkeit, Beistand und Unterstützung für Kinder, Jugendliche und junge Erwachsene in und nach gesellschaftlichen Krisen. Deutscher Ethikrat, Berlin. https://www.ethikrat.org/publikationen/ (As at 21.12.2023)

[47] Bundesministerium für Familie, Senioren, Frauen und Jugend (Ed) (2023) Nationaler Aktionsplan „Neue Chancen für Kinder in Deutschland“. BMFSFJ, Referat Öffentlichkeitsarbeit, Berlin. www.bmfsfj.de/bmfsfj/themen/familie/nationaler-aktionsplan-kinderchancen (As at 21.12.2023)

